# Development of Novel Root Canal Obturation Techniques Using Prototype Obturators in Conjunction With a Resin-Based Hybrid Root SEAL (MetaSEAL) Soft Paste Sealer

**DOI:** 10.7759/cureus.80009

**Published:** 2025-03-04

**Authors:** Taisuke Shokaku, Kohei Shimizu, Tomomi Arai, Takuya Yasukawa, Makoto Hayashi, Osamu Takeichi

**Affiliations:** 1 Department of Endodontics, Nihon University School of Dentistry, Tokyo, JPN; 2 Division of Advanced Dental Treatment, Dental Research Center, Nihon University School of Dentistry, Tokyo, JPN

**Keywords:** apical leakage test, hybrid root seal, metaseal soft, metaseal soft paste, prototype obturator, resin sealer, root canal obturation, sealer backfill, sealer obturation, sealer-only backfill

## Abstract

Background: Sealer-dependent single-cone root canal filling methods have recently been widely applied clinically. With the advancement of sealers and obturating instruments, the sealer-only root canal filling method may be established and shifted in the future. In this study, we evaluated the sealing effect of the prototype obturator on various sizes of plastic models and bovine dentin root canal models using only a hybrid root seal without using a core material.

Methods: The study assessed the sealing efficacy of a prototype obturator in plastic models of varying sizes and bovine dentin root canal models. Plastic models (apical diameters: 0.25, 0.35, and 0.45 mm; taper: 0.07; length: 18.5 mm) were filled with MetaSEAL Soft Paste (Sun Medical, Shiga, Japan) and obturated using the prototype obturator, NT condenser (US trade name: Microseal condenser, Kerr Corp., Orange, CA), and JIZAI (MANI INC., Utsunomiya, Japan). Each device was inserted to a depth of 17.0 mm and rotated forward at 500 rpm, but only JIZAI used reverse rotation. After obturation, the models were sectioned into apical, middle, and coronal parts. Void formation and apical leakage were analyzed using a stereoscopic microscope and the image analysis software (n = 14 each, SigmaScan Pro 5.0, Hulinks, Tokyo, Japan). Then, bovine dentin models were prepared with nickel-titanium files, and apical leakage was assessed (n = 24). Statistical analysis used the Kruskal-Wallis test with Dunn's post hoc test (α = 0.05), ensuring 80% power.

Results: The prototype obturator consistently exhibited fewer air voids and reduced dye leakage compared to the NT condenser and JIZAI. In 0.25- and 0.35-mm plastic models, it showed significantly fewer voids and less dye penetration (p < 0.01-0.05). Smaller canals (0.25 and 0.35 mm) had superior sealing performance over 0.45 mm (p < 0.01). In bovine dentin, the prototype obturator demonstrated the best sealing ability, with significantly lower dye leakage than the NT condenser (p < 0.01) and JIZAI (p < 0.05).

Conclusions: These results highlight the prototype obturator's superior sealing and void reduction. Canal size influenced sealing efficacy, emphasizing the need for appropriate instrument size selection. Further studies should assess performance in complex root anatomies and human teeth.

## Introduction

Advances in root canal filling techniques have significantly improved the success rate of endodontic treatment [1]. Numerous effective methods for root canal filling have been introduced, along with various types of sealers, including bioceramics [2,3] and resin [4]. Some studies recommend using sealers to improve sealing [5,6], and Grossman's principles of filling emphasize the use of a combination of solid filling materials [7]. In recent years, the sealer-dependent single-point method has garnered attention and demonstrated favorable clinical outcomes. This method typically employs a gutta-percha cone as a guide [8]. Whereas a high sealer occupancy rate in root canal filling has been reported to increase void formation [9,10], experimental studies have shown that root canal obturation using sealer alone, without a core material, can achieve favorable outcomes [11,12]. Each sealer possesses distinct characteristics. Bioceramics cement sealers provide high adhesion through the expansion effect caused by wetting [2,3,13], while a resin sealer achieves sealing through strong adhesion to the root canal wall [14]. These sealers are generally injected or transported into the root canal using a tip attached to an injection syringe or a gutta-percha point as a single cone used as a guide [5,6]. However, this method causes air voids [9,10], extra plastic waste from the tip used for injection, and problems in terms of cost-effectiveness. The utilization of a transport device represents one method of sealer delivery. Historically, Lentulo spiral fillers (Henry Schein Inc., Melville, NY) were employed, but concerns arose regarding air void inclusion and sealing efficacy [15]. This study focuses on developing a safer and more efficient intracanal sealer delivery device that can be an alternative to the current syringe and point techniques. A previous investigation identified the optimal standard condenser through a sealing test utilizing standard plastic root canal models [16]. Although there are many studies on bioceramics cement-based sealers [2,3], the literature on MetaSEAL Soft and MetaSEAL Soft Paste (also known as hybrid root seal; Sun Medical, Shiga, Japan) is limited [17,18], with a paucity of reports on the filling condition in standardized straight root canal models [16]. This investigation aimed to assess the sealing efficacy of a hybrid root seal in root canal models of varying dimensions when utilizing different condensation instruments for sealer obturation without core material.

## Materials and methods

**Figure 1:**
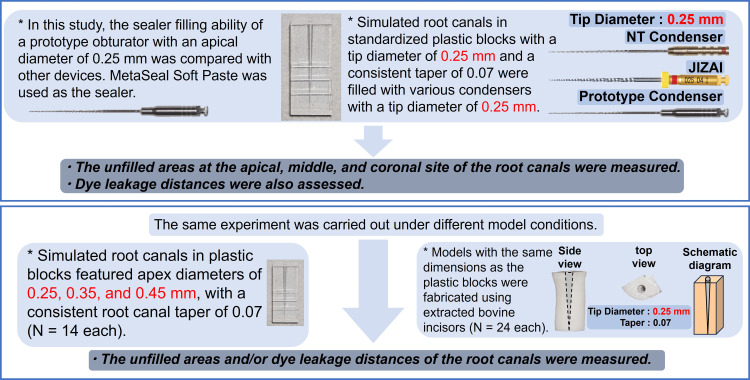
Summary of the present study procedures

Figure 1 shows a schematic summarizing the present study. In this study, we evaluated the sealing effect of the prototype obturator on various sizes of plastic models and bovine dentin root canal models, utilizing only a hybrid root seal without a core material.

Sample size setting

Prior to the initiation of the study, statistical power analyses were performed using G*Power (version 3.1, Heinrich-Heine-Universität Düsseldorf, Düsseldorf, Germany). The study samples were divided into three groups for statistical analysis. Calculations indicated that a minimum sample size of 14 was required to achieve 80% statistical power with a significance level of 0.05 (effect size: 0.5). As a result, a minimum sample size of 14 was adopted.

Standardized plastic root canal model

**Figure 2:**
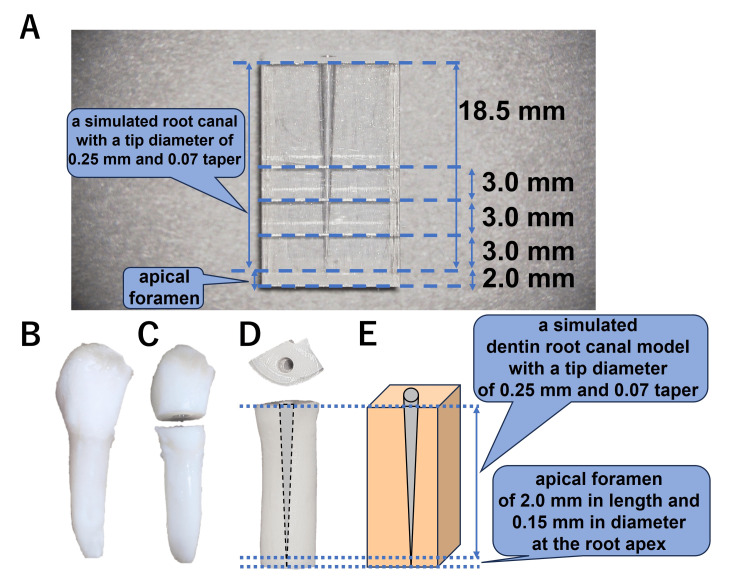
Standardized plastic root canal model and standardized root canal models using dentin walls of extracted bovine incisors. (A) A plastic block with an 18.5-mm working zone is utilized, incorporating a simulated apical foramen. The foramen, measuring 0.15 mm in diameter, is situated 2.0 mm below the apex. (B,C) The tooth is sectioned into a crown and root at the cementoenamel junction. (D,E) A simulated dentin root canal model with a tip diameter of 0.25 mm, 0.07 taper, and an apical foramen of 2.0 mm in length and 0.15 mm in diameter at the root apex, created by the bovine root canal wall, is formed

In this study, using extracted teeth was considered more appropriate; however, due to the emphasis on conservative treatments in Japan in recent years, tooth extractions have been minimized, making it very difficult to collect extracted teeth of the appropriate size. Considering the early experimental nature of this investigation, we determined that utilizing plastic models rather than extracted teeth would be more appropriate. This decision was made to gather preliminary data from standardized models, facilitating the advancement of the study. By employing plastic models at this initial stage, we can establish a foundation for further research progression. A stainless-steel rod (MANI INC., Utsunomiya, Japan) was utilized as a central rod in producing the plastic block, which was designed to create simulated single straight root canals (Nisshin Dental Products, Kyoto, Japan). The smooth-surfaced rods featured tip diameters of 0.25, 0.35, and 0.45 mm, with a consistent root canal taper of 0.07. A plastic block with an 18.5-mm root canal length was utilized, incorporating a simulated apical foramen. The foramen, measuring 0.15 mm in diameter, was situated 2.0 mm below the tip of the root canal (Figure 2A) [16].

Root canal filling using various condensation instruments

In this study, we used a prototype condenser with a pitch angle of 11°, a pitch number 22, and a tip diameter of 0.25 mm with a 0.02 constant taper based on our previous results [16]. The standardized plastic root canal model was positioned vertically and secured using a vice [16]. A locking apparatus with an up-and-down motion was utilized to mount a 16:1 contra-angled handpiece (ATR Tecnika Vision Motor, Dentsply Maillefer, Tulsa, OK). The prototype obturator and MetaSEAL Soft Paste were utilized to fill the root canals within the plastic block. The sealer, which was mixed on a mixing paper pad before application, was applied evenly to the condenser surface while rotating each condenser at 200 rpm. The insertion length of the obturator was set to 17.0 mm, 1.5 mm shorter than the tip of the root canal, and the obturator was moved with an up-and-down motion within a 17.0-mm range at a speed of 500 rpm, repeating the motion four times [16]. As with the prototype obturator, the NT condenser (US trade name: Microseal condenser, Kerr Corp., Orange, CA) and JIZAI (MANI INC., Utsunomiya, Japan) were used to fill the root canal. The tip diameter of these instruments was 0.25 mm. The JIZAI was used to fill the root canal with counterrotation.

Evaluation of unfilled area following root canal filling

After filling the root canals with each instrument, the plastic models were stored in a constant temperature bath at 37°C under conditions of sufficient humidity for one week, and the root canal-filling status of the plastic models of each size was evaluated. The plastic block was divided into three sections: apical, middle, and coronal, at distances of 3, 6, and 9 mm from the root canal tip, respectively. Sectioning was performed using a slow-speed saw (IsoMet; 11-1180-170; Buehler, Lake Bluff, IL) equipped with a thin diamond disk (4-inch diamond cutting wheel, Buehler, Lake Bluff, IL), operated under constant water cooling. A stereoscopic microscope (SMZ18; Objective lens P2-SHR Plan Apo 2x; Nikon, Tokyo, Japan) and a Complementary Metal-Oxide Semiconductor Camera (CMOS; Digital Sight 1000; Nikon, Tokyo, Japan) were utilized to capture and analyze images of the surface area. Images were analyzed utilizing the image analysis software (SigmaScan Pro 5.0; Hulinks, Tokyo, Japan), and the areas before obturation and the areas exhibiting void formation after obturation were quantified in square millimeters.

Assessment of apical leakage following root canal filling

Apical leakage was evaluated using the dye penetration test to evaluate the apical sealing ability of each size of the plastic model after root canal filling with each condensation instrument. The tips of all specimens were immersed in a 2% methylene blue solution (methylene blue trihydrate; Hayashi Pure Chemical Ind., Ltd., Osaka, Japan) for 48 hours and then rinsed with distilled water. A low-speed saw was used to cut the roots longitudinally along their buccolingual axis under constant water cooling. The extent of methylene blue dye penetration from the root apex was measured as apical leakage. The leakage distance of methylene blue was measured and evaluated using a stereomicroscope and a CMOS Camera. The photographs were scanned, and measurements were calculated and converted to millimeters using the image analysis software.

Fabrication of standardized root canal models using dentin walls of extracted bovine incisors

**Figure 3:**
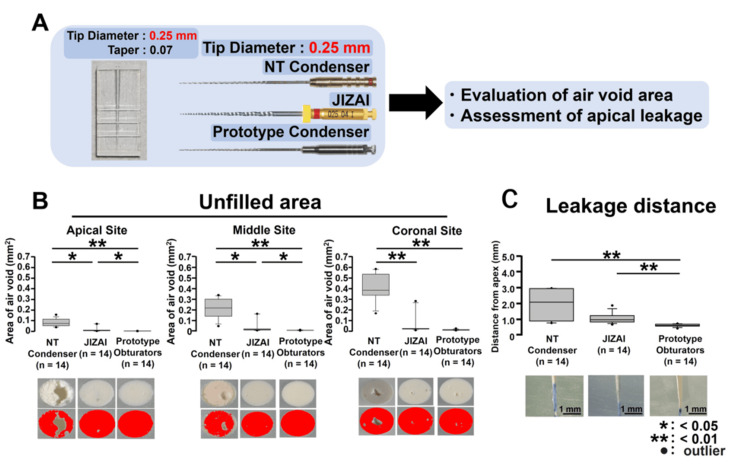
Apical leakage test after filling in 0.25-mm tip diameter plastic simulated root canal models using various 0.25-mm tip diameter obturators. (A) To assess the differences in filling ability among the NT condenser, prototype obturator, and JIZAI groups, the air void area and apical dye-leakage distance were measured after filling 0.25-mm simulated root canal models with various obturators with diameters of 0.25 mm. (B) Air void area after filling. (C) Apical dye-leakage distance after filling

**Figure 4:**
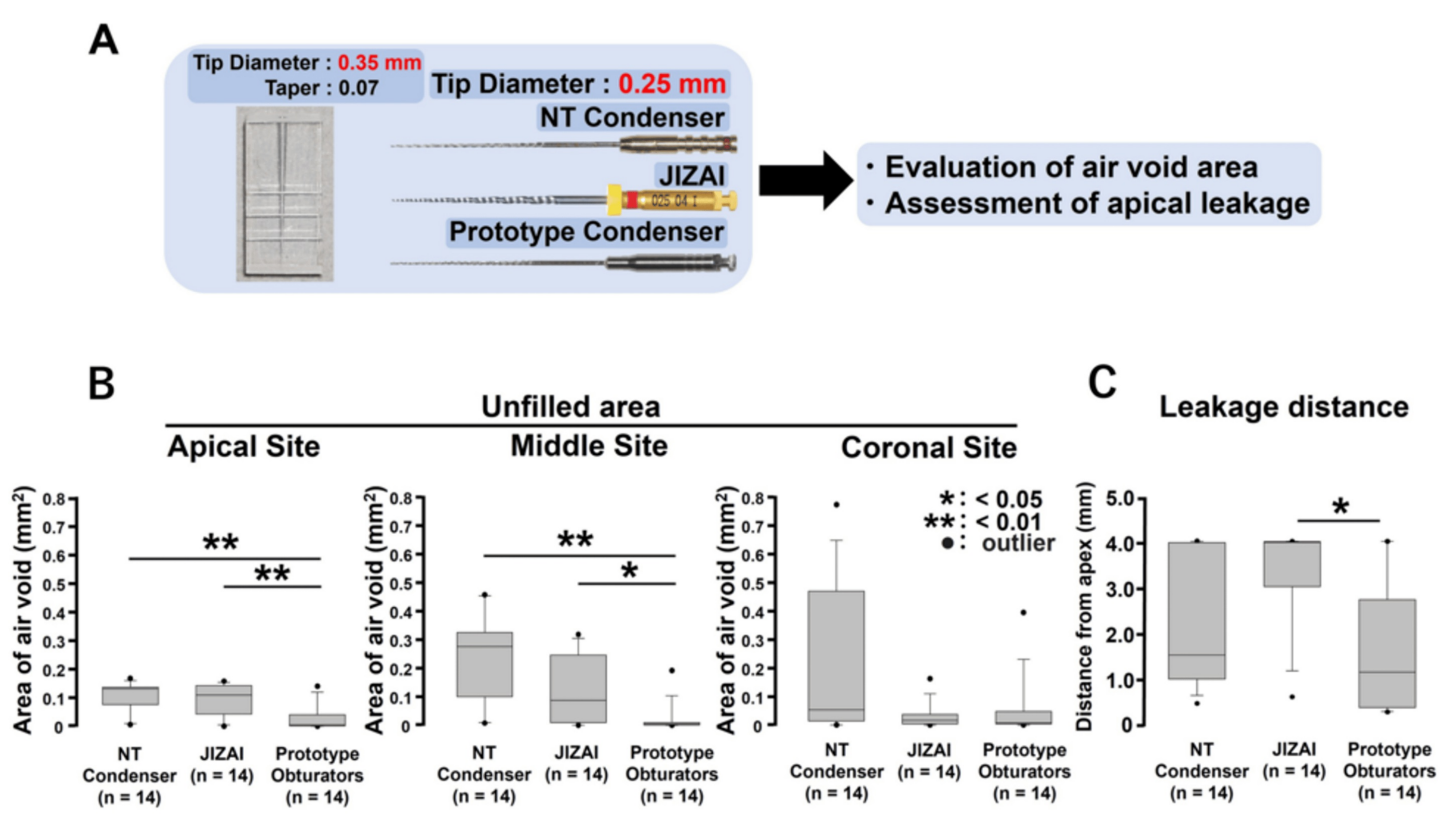
Evaluation of root canal filling in a 0.35-mm tip diameter plastic simulated root canal model with various 0.25-mm tip diameter obturators. (A) To evaluate the differences in filling ability among the NT condenser, JIZAI, and prototype obturator groups when the root canal size was enlarged, the air void area and apical dye-leakage distance were measured after filling a 0.35-mm simulated root canal model with various obturators with a diameter of 0.25 mm. (B) Air void area after filling. (C) Apical dye-leakage distance after filling

**Figure 5:**
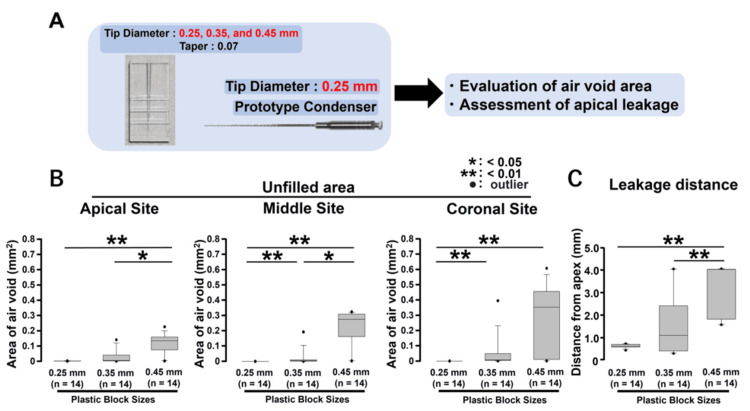
Evaluation of the dependency of prototype endodontic obturator performance (tip diameter 0.25 mm) on root canal size (tip diameters 0.25, 0.35, and 0.45 mm). (A) To investigate the effect of an obturator with a tip diameter of 0.25 mm on filling performance and apical sealing in models of different sizes, the tip diameters of the models were set to 0.25, 0.35, and 0.45 mm, and the change in filling performance was analyzed. (B) Air void area after filling. (C) Apical dye-leakage distance after filling

**Figure 6:**
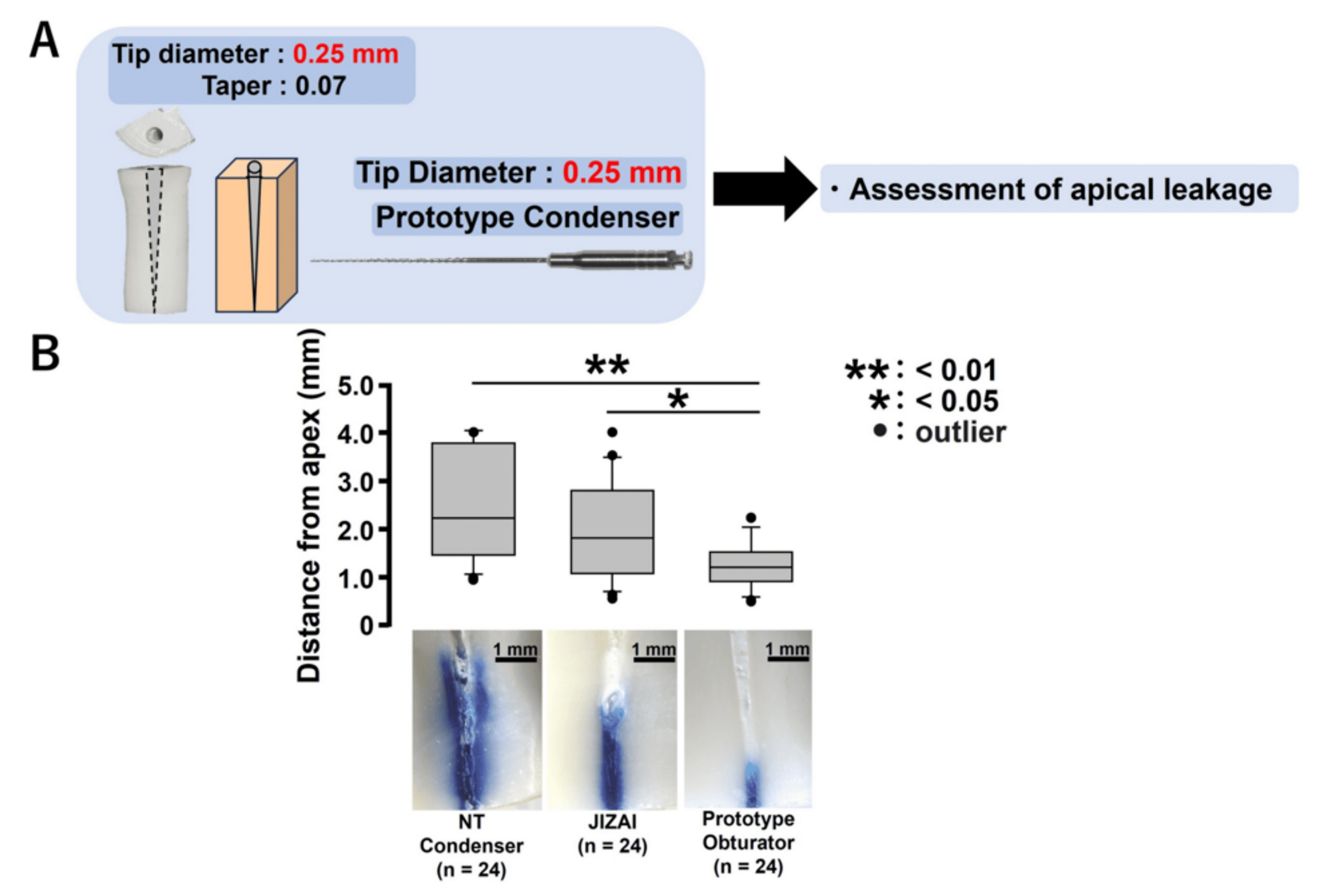
Apical leakage test in standardized root canal models (tip diameter 0.25 mm) made from dentin walls of extracted bovine incisors. (A) To verify the reproducibility of previous results obtained using a plastic root canal model, apical leakage tests were performed after root canal filling with various obturators (tip diameter 0.25 mm) using a standardized dentin root canal model with a tip diameter of 0.25 mm and a 0.07 taper. (B) Apical dye-leakage distance after filling

As described above, collecting a fixed number of human-extracted teeth is difficult and time-consuming (see Standardized Plastic Root Canal Model in the Materials and Methods section). Thus, to confirm the reproducibility of the results obtained with the plastic root canal simulation, dentin models with the same dimensions as the plastic root canal models were fabricated using extracted bovine incisors according to a previously reported method [19]. Initially, soft tissues, including the periodontal ligament surrounding the root, were physically removed. Using a low-speed saw, the tooth was then sectioned into crown and root at the cementoenamel junction (Figures 2B, 2C). The sites used to evaluate the plastic model were 3.0, 6.0, and 9.0 mm from the top of the apical root foramen located 2.0 mm wide from the bottom of the model. To incorporate these lengths and provide a margin, a dentin piece approximately 13.0 mm in length was used to create the dentin standard root canal model. The root was cut with a length of approximately 13 mm and further divided into two equal parts parallel to the tooth axis (Figure 2D). This procedure created two simulated root canal specimens from a single extracted bovine incisor. Subsequently, a 2.0-mm cavity was created in the dentin at the center of the cross-section, parallel to the tooth axis, to facilitate the engagement of the nickel-titanium file. A round steel bur (∅005, DENDIA GmbH, Feldkirch, Austria) and a 005 end bur (∅005, Angelus Japan, Osaka, Japan) were used to create a 2.0-mm cavity in a cross-sectional plane of the crown side as the upper surface. A nickel-titanium rotary file (tip diameter 0.20 and 0.25/07 tapered RE file one, YOSHIDA, Tokyo, Japan) was then used to prepare the root canal starting from the 2.0-mm cavity and extending 2.0 mm above the base of the dentin block. The remaining 2 mm of dentin from the tip of the prepared root canal to the base was penetrated using a #15 K-file (MANI INC., Utsunomiya, Japan). This resulted in a simulated dentin root canal model with a tip diameter of 0.25 mm, 0.07 taper, and an apical foramen of 2.0 mm in length and 0.15 mm in diameter, created by the bovine root canal wall (Figures 2D, 2E). A lubricant (RC-Prep, Premier, Plymouth Meeting, PA) was applied during instrument manipulation. The tooth was cleaned with a 3% sodium hypochlorite solution (NISHIKA, Yamaguchi, Japan) and finally rinsed with distilled water. Based on previous reports [16] and the data obtained in this preliminary study, it was found that when performing root canal filling with a simulated root canal plastic block with an apical diameter of 0.25 mm, the optimal filling rate was achieved when using a similarly sized prototype condenser with an apical diameter of 0.25 mm (Figures 3-5). Thus, in a present preliminary experiment using a bovine tooth as a simulated root canal model, a simulated apical foramen 2.0 mm in length and 0.15 mm in diameter was created at the tip. From there, a root canal with a tip diameter of 0.25 mm and a taper of 07 was fabricated. For root canal filling, the respective filling instruments with an apical diameter of 0.25 mm were utilized consistently for comparison (Figure 6).

Statistical analyses

As the Shapiro-Wilk test revealed a nonnormal distribution of the data, nonparametric methods were applied. The Kruskal-Wallis test was used for group comparisons, followed by Dunn’s post hoc test for pairwise comparisons. Statistical analyses were conducted using GraphPad Prism, version 10 (GraphPad Software, Boston, MA), with a significance level set at α = 0.05 (p < 0.05) for all tests performed.

## Results

Unfilled areas after filling in 0.25-mm tip diameter plastic simulated root canal models using various 0.25-mm tip diameter obturators

A schematic of this experimental procedure is presented in Figure 3A. Examination of the cut surfaces in the apical, middle, and coronal areas showed that the JIZAI and prototype obturator groups had significantly smaller air void regions compared to the NT condenser group across all areas (Figure 3B; vs. JIZAI: apical and middle areas: p < 0.05, coronal area: p < 0.01; vs. prototype obturator: apical, middle, and coronal areas: p < 0.01). Additionally, the prototype obturator group exhibited significantly less air void area in both the apical and middle regions compared to the JIZAI group (Figure 3B, p < 0.05) (n = 14 for each group).

Apical leakage test after filling in 0.25-mm tip diameter plastic simulated root canal models using various 0.25-mm tip diameter obturators

The apical sealing ability evaluation, conducted through an apical leakage test, demonstrated that the prototype endodontic obturator group exhibited a significant reduction of dye-leakage distance compared to the NT and JIZAI groups (Figure 3C, p < 0.01) (n = 14, respectively).

Evaluation of root canal filling in a 0.35-mm tip diameter plastic simulated root canal model with various 0.25-mm tip diameter obturators

A schematic of this experimental procedure is presented in Figure 4A. The prototype obturator group exhibited a significantly reduced air void area compared to the NT condenser and JIZAI groups at the apical and middle regions (Figure 4B, vs. NT condenser: apical and middle areas; p < 0.01, vs. JIZAI; apical area: p < 0.01, middle areas: p < 0.05). Additionally, the prototype obturator group demonstrated a considerably shorter distance of apical dye leakage than the JIZAI group (Figure 4C, p < 0.01) (n = 14, respectively).

Evaluation of the dependency of prototype endodontic obturator performance (tip diameter 0.25 mm) on root canal size (tip diameters 0.25, 0.35, and 0.45 mm)

Our results revealed that using a prototype obturator with a tip diameter of 0.25 mm in a plastic model with a tip diameter of 0.25 or 0.35 mm provided good filling performance and apical sealing (Figures 3, 4). To further investigate the effect of an obturator with a tip diameter of 0.25 mm on filling performance and apical sealing in models of different sizes, we set the tip diameters of the models to 0.25, 0.35, and 0.45 mm and analyzed the change in filling performance (Figure 5A). Analysis of the unfilled air void area at the cut surface after obturation revealed that the 0.25-mm group had significantly smaller air void areas at the apical, middle, and coronal regions than the 0.45-mm group (Figure 5B, p < 0.01). The 0.35-mm group also had significantly smaller air void areas at the apical and middle regions than the 0.45-mm group (Figure 5B, p < 0.05). Moreover, the void area in the 0.25-mm group was significantly smaller than that observed in the 0.35-mm group in the middle and coronal regions (Figure 5B, p < 0.01). Regarding the apical dye-leakage test, both the 0.25- and 0.35-mm groups showed significantly shorter distances than the 0.45-mm group (Figure 5C, p < 0.01) (n = 14, respectively).

Apical leakage test in standardized root canal models (tip diameter 0.25 mm) fabricated using dentin walls of extracted bovine incisors

A schematic of this experimental procedure is presented in Figure 6A. The experimental results demonstrated that the dye-leakage distance in the apical region was significantly reduced in the group utilizing the prototype obturator compared to the groups employing the NT condenser (p < 0.01) and JIZAI (p < 0.05) (Figure 6B, n = 24, respectively).

## Discussion

Previous studies have suggested that root canal filling with sealer alone could be an effective and direct method of root canal filling [20]. However, establishing this method requires the development of instruments that can transport the sealer to every corner of the root canal. Therefore, in the present study, we investigated the sealing efficacy for obturation of a prototype condenser based on previously established standards in a model in which the root canal was sealed solely with a root canal sealer without using a core material. This investigation yields novel findings, as no comparable studies are currently available in existing literature. Thus, in the present study, the obturating condition and apical leakage condition were examined in detail using plastic models of various root apex sizes, employing both a prototype condenser (MANI INC., Utsunomiya, Japan) and a commercially available condenser and instrument. In recent years in Japan, due to the difficulty of obtaining human-extracted teeth and the variability in root canal size, studies have shown that an effective approach is to use the dentin portion of bovine teeth to simulate the dentin wall and create a standardized root canal model [19]. Therefore, to confirm the reproducibility of the results obtained with the plastic model, we also examined the leakage condition using a root canal model that simulated the dentin wall. Since actual root canals have complex shapes, such as curvatures, isthmuses, and fins, applying the obturators directly to natural teeth may result in inconsistent conditions and standards. Therefore, a simple straight plastic model was first used to fill the root canal with sealer using the NT condenser, JIZAI reverse rotation, and the prototype condenser, and the filling condition was investigated as a preliminary experiment. Then, reproducibility was verified using a root canal model simulating the dentin wall created using an extracted bovine anterior tooth.

Comparative analysis of prototype obturator and conventional instruments for sealer-only root canal filling

The primary objective of endodontic treatment is to achieve thorough cleaning, shaping, and hermetic sealing of the root canal system to prevent reinfection [1]. The presence of voids and gaps in root canal fillings can serve as potential sites for bacterial colonization, thereby compromising the success of the treatment [21]. This study employed a simulated root canal model (tip diameter: 0.25 mm, taper: 0.07) to evaluate the extent of air void formation following obturation with MetaSEAL Soft Paste using different filling instruments. Three instruments were tested: the NT condenser, the JIZAI instrument with reverse rotation, and a prototype obturator, all precisely matched to the canal size (tip diameter: 0.25 mm; Figure 3A). The results indicated that the prototype obturator significantly minimized air void formation compared to the other instruments, demonstrating superior sealing efficacy in straight root canals (Figure 3B). The prototype obturator exhibited superior filling capabilities, even in root canals with an apical diameter of 0.35 mm, which exceeded the condenser tip's diameter of 0.25 mm. The prototype obturator effectively reduced air void formation in both the apical and middle sections of the root canal compared to conventional obturation instruments, following obturation with MetaSEAL Soft Paste (Figures 4A, 4B). Previous studies have reported that the highest filling rate for transporting calcium hydroxide formulations into C-shaped root canals was achieved using a Lentulo spiral filler and a syringe system. This is thought to be because the rotation of the Lentulo spiral filler removes the small air bubbles present in the root canal, allowing the calcium hydroxide formulation to diffuse into the irregular canal canals and fins of the C-shaped canal, allowing better distribution and contact of calcium hydroxide to the inner surface of the root canal [22]. Similar to this report, the improved sealing of the prototype obturator may be attributed to its structural design and functional characteristics, which contribute to the uniform distribution of filler material and the suppression of unfilled voids. Conversely, the JIZAI obturator and NT condenser, both with a 0.25 mm tip diameter, exhibited limitations in adequately compacting the filling material within wider root canals. As a result, the prototype obturator not only suppressed unfilled void formation but also improved the density and homogeneity of the filling material in the apical and middle regions. This study is the first to demonstrate the efficacy of a prototype obturator in larger root canals and highlights the need for further investigations to assess its performance across varying root canal morphologies and over extended observation periods.

Influence of root canal size on prototype obturator filling performance

The review article introduces that several researchers have shown that the state of root canal preparation affects the outcome of root canal filling [23]. Thus, this study further investigates the influence of root canal size on the performance of a prototype endodontic obturator with a tip diameter of 0.25 mm. Using plastic root canal models with apical diameters of 0.25, 0.35, and 0.45 mm, the findings revealed relationships between the dimensions of the root canal and the filling properties of the obturator. Larger root canals exhibited increased air void areas due to insufficient pressure application during obturation, leading to uneven material distribution and reduced sealing performance. The size of the prepared root canal and the size of the instruments used for root canal filling influence the results of root canal filling [1,23]. The present findings highlight the necessity of using a condenser that matches the root canal size to ensure optimal filling. MetaSEAL Soft Paste, known for its high fluidity and adhesiveness, requires appropriate pressure application to prevent air void formation and improve adaptation to canal walls. Future research should verify these results using complex root canal morphologies, such as shovel-shaped roots, isthmuses, fins, and curved root canals [22]. Furthermore, developing obturators adaptable to various root canal sizes remains a crucial challenge. This study provides valuable insights into the relationship between root canal size and obturator performance, emphasizing the importance of appropriate obturator selection to enhance sealing performance and improve the success rate of root canal treatment.

Critical evaluation of dye-leakage tests and validation of plastic model findings in bovine dentin models

This study critically evaluated dye-leakage tests and validated the findings obtained from plastic root canal models using bovine dentin models, focusing on the apical sealing ability of a prototype endodontic obturator in simulated root canal environments. Apical sealing is a crucial factor in root canal treatment success, influenced by sealer composition, canal preparation and filling techniques, and the presence of a smear layer [24-26]. Various evaluation methods, including bacterial penetration tests, radioisotope methods, dye-leakage tests, fluid filtration methods, and electrochemical approaches [27-31], have been developed, with the dye-leakage test widely used for its simplicity and reproducibility [32,33]. Although these tests should be employed in future studies, this is a preliminary study, and the dye penetration test was employed first to search for root apex sealing properties more easily. The prototype obturator achieved significantly better apical sealing performance in the dye-leakage test than the other groups. Its design may have effectively promoted adhesion and minimized apical microleakage by uniformly spreading the filling material to the root canal wall, similar to previous studies using the Lentulo spiral filler [22]. In addition, experiments using obturators with different root canal diameters showed that the prototype obturator had better sealing performance than the JIZAI. Furthermore, as the apical root canal diameter increased compared with the size of the prototype obturator, the apical leakage after root canal filling also increased accordingly. This result may be related to reports that the size of the preparated root canal affects the outcome of the obturation procedure [23]. However, because this study used a straight plastic root canal model, it was found that it is necessary to verify it with models of various shapes and curvatures that are more representative of the clinical situation of curved or branched root canals in the future. A standard root canal model was created using extracted bovine incisor dentin blocks to verify these results in a more clinically relevant dentin environment. Root canal filling and apical leakage tests using MetaSEAL soft paste confirmed that the prototype obturator had superior sealing performance compared with the NT condenser and JIZAI reverse rotation. This suggests that the prototype obturator may improve the distribution and adhesion of materials to the dentin surface and ensure uniform compaction of the sealer, similar to the report using the existing instrument, the Lentulo spiral filler [22]. Unlike plastic models, the inner surface of the root canal of dentin models is structurally porous, which affects the flow and adhesion of the sealer more. However, because bovine and human dentin have different structural properties, the sealer adhesion observed in this study may not be maintained in human teeth [34]. Previous reports indicated that the difference in the number of dentin tubules or their diameters is a factor affecting the adhesive strength of dentin adhesion, and a comparison between bovine and human dentin has been investigated [35,36]. The report indicated that the structure of dentin is similar to that of human wisdom teeth and bovine premolars [35]. In addition, a comparison of the organic substrates of the dentin showed that human teeth and bovine teeth are similar [36]. As indicated by these reports, the use of bovine dentin as a substitute for human dentin is reasonable, and stability has been achieved with respect to its use [37]. Therefore, the data obtained with the bovine dentin model will play an important role in its application to human teeth. However, since experiments using human teeth are essential, we plan to apply the data obtained with the bovine tooth model in the next study plan. Future studies should focus on optimizing the design of the prototype obturator, examining its interaction with various filling materials, and verifying its performance in complex and curved root canal structures. This can contribute to further improvements and potential clinical applications.

Limitations of the present study

One limitation of this study is that the evaluation of the sealing ability of sealer-based root canal filling materials was based solely on the void area and root apex stain leakage distance. Although void volume was evaluated using stereomicroscopy, a more comprehensive, nondestructive approach, such as microcomputed tomography, is recommended to evaluate the entire root canal from the crown to the apex [38]. Furthermore, some studies have questioned the reliability of the dye-leakage test in assessing root apical sealing capacity [39]. Therefore, future studies should incorporate more appropriate methods for evaluating apical sealing capacities, such as bacterial penetration tests, radioisotope methods, fluid filtration methods, and electrochemical methods. Structural similarities between bovine and human teeth have been reported [35-37]. However, further studies using human-extracted teeth are needed to finally validate these findings and bring them closer to a clinical environment. In addition, the prototype condenser used in this study is made of stainless steel and cannot be used for curved root canals. Therefore, the present preliminary experiments were performed only on straight root canals to verify the usefulness of our previously obtained conditions: pitch angle of 11°, pitch number of 22, and tip diameter of 0.25 mm with a 0.02 constant taper. Therefore, we plan to make a prototype nickel-titanium condenser based on the standard obtained in this study and examine its application to curved root canals.

## Conclusions

In this study, the root canal filling performance of the prototype obturator was evaluated using a plastic model and a bovine dentin model. The prototype obturator demonstrated significantly better apical sealing performance than the other instruments, along with shortened apical leakage distance and reduced formation of unfilled air voids. It also became clear that selecting an instrument based on root canal size is crucial. The results of this study indicate that further improvements to the prototype obturator could enhance its performance. However, the conclusions should be more cautiously framed, emphasizing that the findings are model-based and require further validation in human teeth and complex canal systems. In the future, it will be important to verify the performance of this technology on extracted human teeth with complex root canal shapes and curvatures, as well as to evaluate it through comparative studies with root canal filling methods using various filling materials, such as root canal filling with single gutta-percha cones.
